# Integrating a DNA Strand Displacement Reaction with a Whispering Gallery Mode Sensor for Label-Free Mercury (II) Ion Detection

**DOI:** 10.3390/s16081197

**Published:** 2016-07-29

**Authors:** Fengchi Wu, Yuqiang Wu, Zhongwei Niu, Frank Vollmer

**Affiliations:** 1 Technical Institute of Physics and Chemistry, Chinese Academy of Sciences, Beijing 100190, China; wufengchi@mail.ipc.ac.cn (F.W.); niu@mail.ipc.ac.cn (Z.N.); 2 University of Chinese Academy of Sciences, Beijing 100080, China; 3 Laboratory of Nanophotonics & Biosensing, Max Planck Institute for the Science of Light, Erlangen D-91058, Germany; yuqiang.wu@gmail.com; 4 Division of Biomedical Engineering, Brigham and Women’s Hospital, Harvard Medical School, Boston, MA 02115, USA

**Keywords:** whispering gallery mode, DNA strand displacement, Mercury (II) ions detection, biosensing, label-free detection, optical microcavity, DNA nanotechnology

## Abstract

Mercury is an extremely toxic chemical pollutant of our environment. It has attracted the world’s attention due to its high mobility and the ease with which it accumulates in organisms. Sensitive devices and methods specific for detecting mercury ions are, hence, in great need. Here, we have integrated a DNA strand displacement reaction with a whispering gallery mode (WGM) sensor for demonstrating the detection of Hg^2+^ ions. Our approach relies on the displacement of a DNA hairpin structure, which forms after the binding of mercury ions to an aptamer DNA sequence. The strand displacement reaction of the DNA aptamer provides highly specific and quantitative means for determining the mercury ion concentration on a label-free WGM sensor platform. Our approach also shows the possibility for manipulating the kinetics of a strand displacement reaction with specific ionic species.

## 1. Introduction

Although a number of important techniques have been reported for Hg^2+^ detection, such as those based on colorimetry and fluorescence [1,2], nanoparticles [3] or functional polymers [4], these established methods often have drawbacks due to time-consuming assay procedures, complex sample preparations and requirements for specific laboratory equipment. Therefore, methods and technologies for building sensor devices with high sensitivity and selectivity for facile real-time mercury ion detection are still under investigation. Real-time sensor devices that work in a label-free fashion have received particular attention in recent years since they can overcome the problems associated with the introduction of fluorophores or radiolabels [5,6]. Label-free detection strategies have the additional advantage that they can be more easily integrated on sensor devices, where different transducers are available for readout in the electrical [7,8], mechanical [9,10], and optical domain [11,12]. Among these systems, optical whispering gallery mode (WGM) sensors are an emerging technology platform [13,14] for the label-free detection of a large variety of analytes including virus [15,16], oligonucleotide [17,18], protein [19,20], nanoparticle [21,22], and small molecule [23,24]. WGM sensors transduce the binding of target analytes into a resonance frequency/wavelength shift, thereby sensing the small variations of the effective refractive index in the immediate surroundings of the optical microcavity. Hanumegowda et al. [25] have used a WGM sensor for detecting mercury (II) ions, reporting a limit of detection ~240 nM. Ultra-high sensitivity WGM biosensing has been demonstrated with plasmonic enhancements, achieving a detection limit down to single DNA molecules [26].

In the context of WGM biosensing, DNA strand displacement reactions have been introduced only recently to improve detection limits as well as specificity in bulk nucleic acid detection [27]. DNA strand displacement reactions, in which strands with partial or full complementarity hybridize, thereby displacing pre-hybridized strands [28], have already been used to realize a variety of molecular systems: for controlling DNA self-assembly [29], non-covalent DNA catalysis [30], and generating autonomous DNA nanomachines [31].

Here, we have used a DNA strand displacement reaction that we have integrated with a label-free WGM sensor for demonstrating specific Hg^2+^ ion detection. Our proof-of-principle study allowed us to directly observe a low molecular weight ionic species after binding to a DNA aptamer by recording shifts in the WGM sensor signal. We have quantitated the concentration of mercury Hg^2+^ ions in well-defined sample solutions by analyzing the kinetics of the WGM sensor response, and harnessing the selectivity of the aptamer for demonstrating the potential of highly specific Hg^2+^ detection. 

## 2. Sensor System Design

### 2.1. WGM Sensor

**Figure 1:**
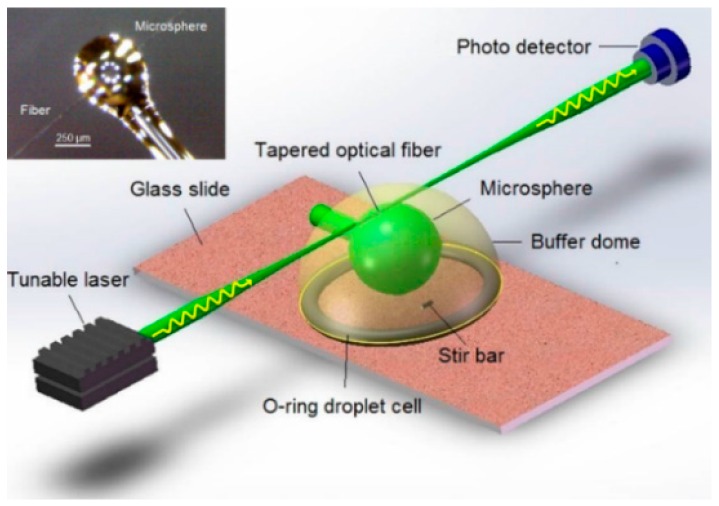
Experimental setup of the WGM sensing platform. The inserted figure displays a micrograph of a microsphere WGM sensor coupled to a tapered optical fiber.

A schematic of the experimental setup is shown in Figure 1. Light from a tunable distributed feedback laser (DFB laser operating at ~1550 nm wavelength, mounted on LDM 4980, ILX Lightwave, Bozeman, MT, USA) was evanescently coupled into a microsphere resonator via a tapered optical fiber (SMF-28e, Corning Inc., Corning, NY, USA), exciting a WGM resonance [27]. The microspheres were ~300 µm in diameter, and made by melting a single-mode optical fiber with an oxygen-butane flame. The microsphere was further functionalized with oligonucleotides (see Experimental Methods section). An O-ring droplet cell was used for immersing the microsphere in ~400 µL of buffer solution, and a micro-stir bar was used for homogenizing the sample cell. The output light of the fiber-coupled microsphere system was monitored by a photo detector, and the WGM spectra of the microsphere were acquired by swept-wavelength scanning of the laser source [32]. The resonance wavelength shift of the WGM spectra due to DNA interactions at the sensor surface was quantitated in units of DNA mass loading (unloading) per millimeter-squared sensor area, pg∙mm^−2^, according to [27]:

(1) $$massloading=\frac{\Delta \lambda }{\lambda }\frac{\left(n_{s}^{2}-n_{m}^{2}\right)R}{2n_{m}\cdot dn/dc}$$

where Δλ was the shift of the resonance wavelength, λ was the nominal wavelength of the DFB laser (here λ ~ 1550 nm), *n_s_* = 1.46 and *n_m_* = 1.33 were the refractive indices of the microsphere and of the aqueous medium, respectively, R was the approximate radius of the microsphere as it was determined by microscopic imaging (here R = ~150 µm), and *dn*/*dc* ≈ 0.166 × 10^−9^ (mm^3^∙pg^−1^) was the approximate incremental refractive index change of a DNA solution. 

### 2.2. Mechanism of Hg^2+^ Detection by Strand Displacement

**Figure 2:**
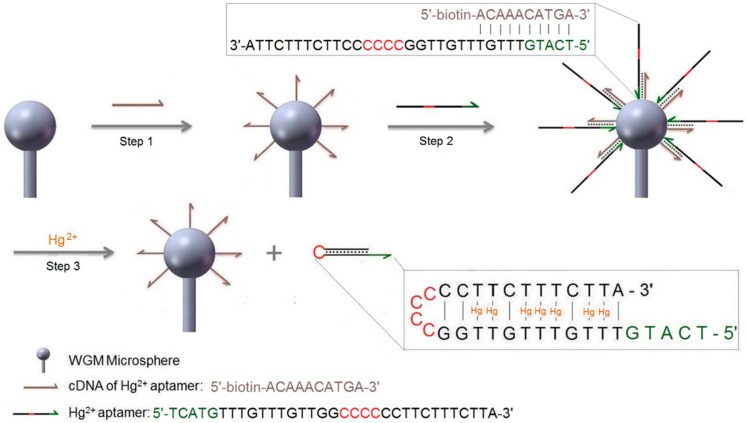
Schematic illustration of the strategy for Hg^2+^ ion detection using a DNA aptamer, a strand displacement reaction, and a label-free WGM sensor. Steps for performing the sensing experiments are: Step 1—attachment of cDNA via streptavidin to surface-adsorbed biotinylated dextran layer; for details of this functionalization procedure see Figure 3a. Wash with buffer. Step 2—hybridization of aptamer, also see Figure 3b for details. Wash with buffer. Step 3—detection of Hg^2+^ by strand displacement reaction.

Two oligonucleotide strands were used in this experiment. One strand was the Hg^2+^ aptamer (5′-TCATGTTTGTTTGTTGGCCCCCCTTCTTTCTTA-3′). This particular aptamer sequence was chosen following the works by Lu et al. [1]. As shown in Figure 2, each aptamer contained 15 thymine nucleotides (T), which can bind up to 7 Hg^2+^. The other strand was a 10 mer cDNA fragment (5′-biotin-ACAAACATGA-3′), complementary to the 5′-terminal bases of the Hg^2+^ aptamer. The cDNA was first immobilized to the surface of a microsphere via biotin-streptavidin interaction (Figure 2, step 1, also see Methods section). The Hg^2+^ aptamer was then hybridized to the cDNA through complementary base-pairing (Figure 2, step 2). In the presence of Hg^2+^, the 3′-terminal fragment of the aptamer partially hybridized and displaced the cDNA in binding to 5′-terminal bases of the Hg^2+^ aptamer by the formation of the T-Hg-T complex. This allowed the Hg^2+^ aptamer to fold into a hairpin structure and from where it released from the microsphere (Figure 2, step 3). The completed process represented a DNA strand displacement reaction according to the definition and pertinent examples given by David Yu Zhang [28].

The WGM sensor was used to record various stages of the microsphere surface functionalization, as well as the unloading of the hairpin DNA upon the injection of mercury ions. From the magnitude of the WGM sensor response we determined the grams of nucleic acid mass-unloading per millimeter-squared sensor area in units (pg∙mm^−2^). We conducted three trials for each type of WGM measurement. A different microsphere was functionalized for each of those trials.

## 3. Experimental Methods

### 3.1. Chemicals

Dextran-biotin (70,000 MW) was bought from Marker Gene Technologies, Inc., Eugene, OR, USA, Oligonucleotide strands were synthesized by Eurofins Genomics. Streptavidin was purchased from New England Biolabs (NEB), Ipswich, MA, USA. Tris-EDTA buffer solution (pH 8.0), MgCl_2_, Hg(NO_3_)_2_, CuCl_2_, KCl, NaCl, NiCl_2_, PbN_2_O_6_, ZnCl_2_, and CaCl_2_ were obtained from Sigma Aldrich, St. Louis, MO, USA. All chemicals were of analytical-reagent grade and were used without further purification.

### 3.2. Microsphere Fabrication 

An SMF-28e optical fiber (Corning Inc., Corning, NY, USA) was cut into small pieces with ~4.5 cm in length. Then, the polymeric coating at one end of each fiber was removed by a fiber stripper, followed by cleaning using an acetone-saturated wiper. After that, the flame from a butane-oxygen microtorch was pointed to the tip of the stripped fiber end until it was softened and formed a microsphere.

### 3.3. Microsphere Surface Modification

The freshly prepared microsphere was immediately placed into an air plasma cleaner (PDC-32G, Harrick, Ossining, NY, USA) and oxidized for 5 min. Then, 2 µL of 10 mg/mL biotin-dextran solution was pipetted onto the microsphere forming a small droplet hanging at the tip of the fiber, allowing for the physisorption of dextran onto the microsphere’s surface [26]. Twenty minutes later, the sphere was rinsed in distilled water for 5 min.

### 3.4. Functionalizing the Microsphere for DNA Strand Displacement Reaction

**Figure 3:**
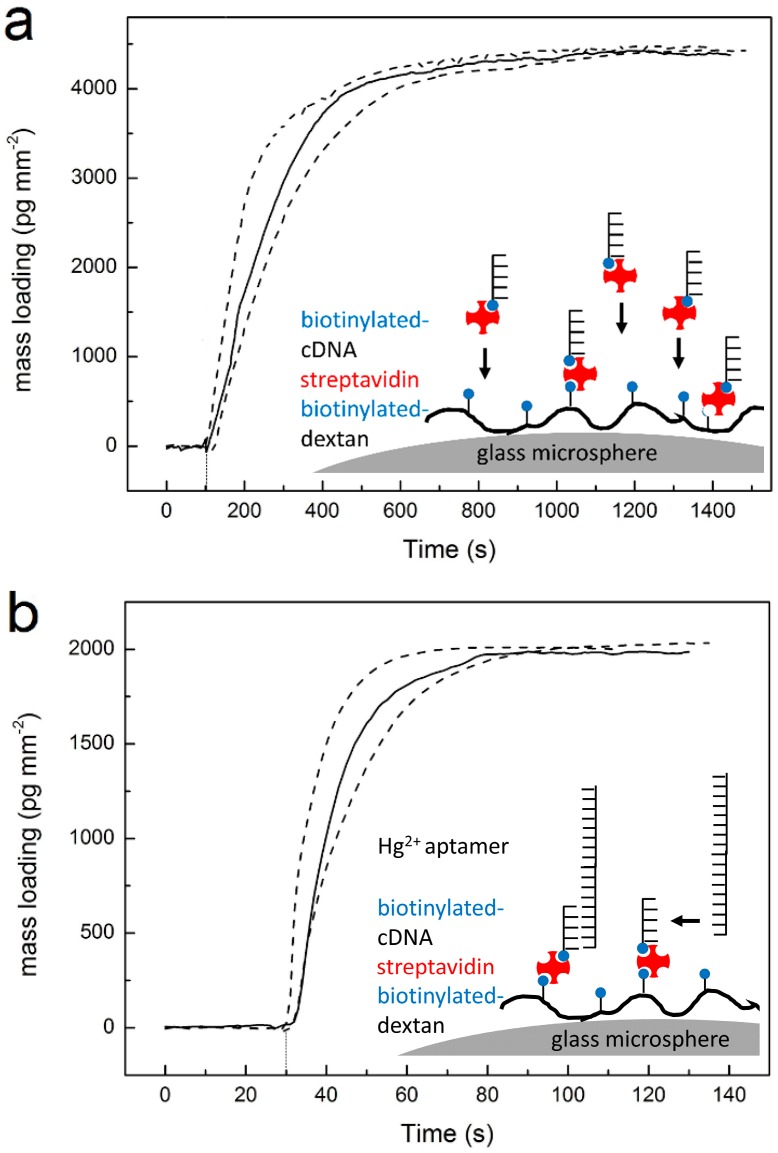
Functionalizing the microsphere for DNA strand displacement reaction. (**a**) WGM mass-loading curve recorded for streptavidin-cDNA complex binding to previously biotin-dextran–modified microsphere; (**b**) WGM mass-loading curve recorded for subsequent hybridization of Hg^2+^ aptamer with cDNA at the microsphere surface. The three curves correspond to three independent experiments performed with different microspheres.

The biotin-dextran modified microsphere was mounted on the sensor system within the droplet cell and then coupled to a tapered fiber for WGM excitation. The microsphere is immersed in 400 µL of pH 8.0 Tris-EDTA-MgCl_2_ buffer (10 mM pH 8.0 Tris-HCl, 1 mM disodium EDTA, and 12.5 mM MgCl_2_) (Figure 1). Then (Figure 2, step 1), 5 μL streptavidin-cDNA mixture (a solution of 24.6 µL of 1 mg/ml streptavidin and 10 µL of 100 µM cDNA) was slowly injecting in the sample cell while stirring the buffer solution. A WGM wavelength shift signal was recorded (Figure 3a), indicating the binding of the streptavidin-cDNA mixture to the biotin-dextran layer on the microsphere. Pre-mixing of streptavidin and cDNA was necessary as initial injection of streptavidin would not result in a high density surface coverage during subsequent cDNA injection as most of the binding sites of streptavidin would then already be occupied by the biotin moieties of the dextran layer [27]. 

The wavelength shift saturated as the biotin-streptavidin interaction equilibrates. At that time, the buffer solution was replaced by 400 µL of pH 8.0 Tris-EDTA-MgCl_2_ buffer. Then, 2 µL of 100 µM Hg^2+^ aptamer stock solution was injected (Figure 2, step 2), upon which a second WGM wavelength shift signal was recorded (Figure 3b), which saturated as the aptamer hybridizes to the cDNA at the microsphere surface. The procedure for surface functionalizing of the microsphere was completed within ~3 min.

### 3.5. Hg^2+^ Detection by Strand Displacement Reaction

**Figure 4:**
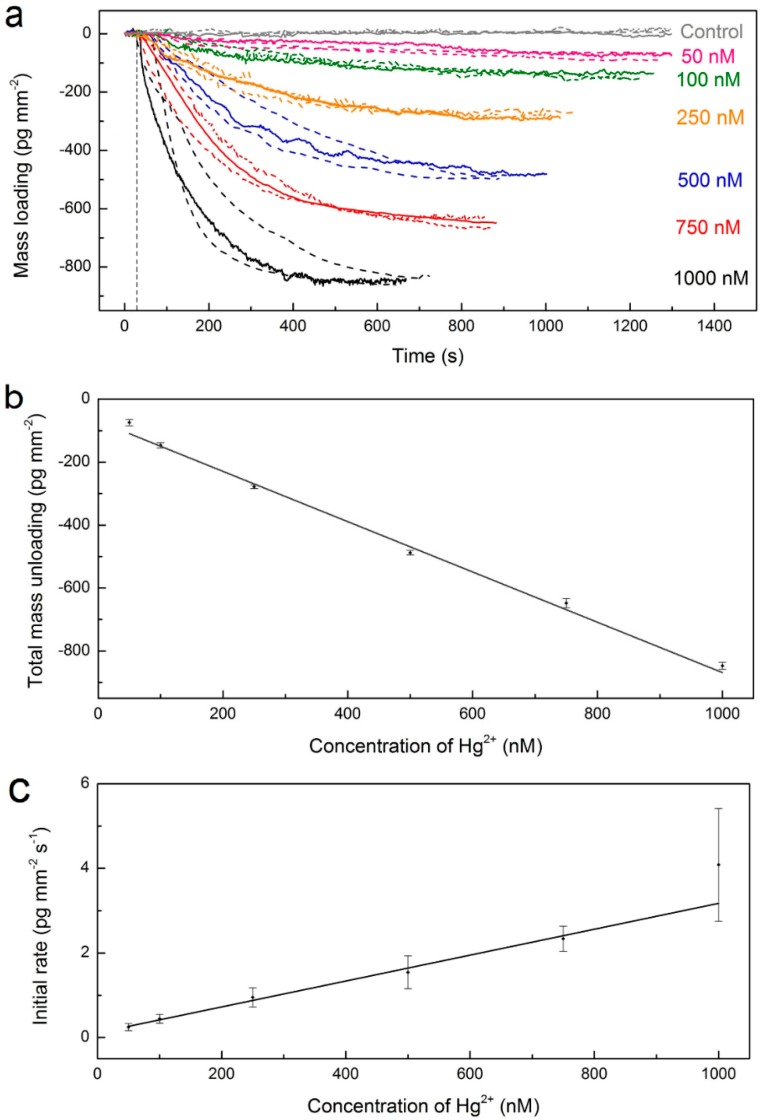
Hg^2+^ detection. (**a**) WGM mass-loading (unloading) curve recorded for different Hg^2+^ concentrations. All the experiments were conducted three times. The parallel results are shown as dashed lines. Vertical lines indicate the time points where the Hg^2+^ solution was injected. The error bar is given by the standard deviation of three experiments performed with different microspheres; (**b**) Linear relationship between the mass-unloading of Hg^2+^ aptamer and the concentration of Hg^2+^; (**c**) Average absolute initial rate of WGM mass-unloading of aptamer as a function of Hg^2+^ concentration.

**Figure 5:**
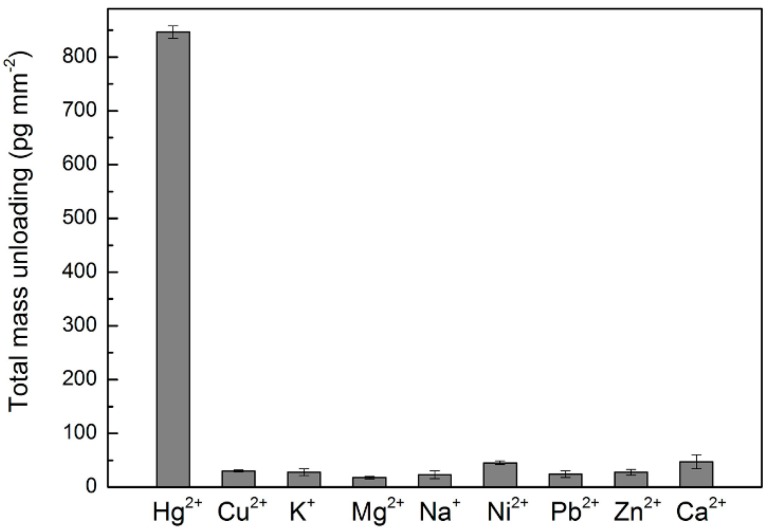
Selectivity of WGM sensor for the detection of Hg^2+^. The concentration of all the metal ions used here was 1 µM. The error bar is given by the standard deviation of three experiments.

The buffer in the sample cell was replaced with pH 8.0 Tris-MgCl_2_ (10 mM pH 8.0 Tris-HCl, and 12.5 mM MgCl_2_) buffer. After waiting for the WGM baseline shift signal to become stable (no significant shift in 5 min), Hg^2+^ solution at specific concentration was added to the chamber (Figure 2, step 3) under slow stirring with the micro stir bar to homogenize the ensuing DNA strand displacement reaction (Figure 4). To test the selectivity of our approach, metal ions including Cu^2+^, K^+^, Mg^2+^, Na^+^, Ni^2+^, Pb^2+^, Zn^2+^, and Ca^2+^ were assayed similarly (Figure 5), at the concentration of 1 µM. Detection of a specific mercury concentration was conducted three times. For each trial, a new microsphere was functionalized.

## 4. Results 

### 4.1. Preparing the WGM Sensor for the DNA Strand Displacement Reaction

To prepare the functionalized microsphere for the DNA strand displacement reaction, the cDNA of the Hg^2+^ aptamer was first bound to the microsphere surface via biotin-streptavidin interactions. For the attachment of cDNA to the microsphere, biotinylated cDNA was pre-mixed with streptavidin. Note that the biotinylated cDNA was used in a concentration equal to two times the concentration of the streptavidin, so that about two binding sites on each streptavidin remained unoccupied, on average, for binding to the biotinylated-dextran layer. As shown in Figure 3a, after temperature equilibration, 5 µL of the streptavidin-cDNA complex was injected into the reaction chamber, where it bound to the biotin-dextran layer that was previously physisorbed onto the microsphere (also see Experimental Methods Section). The quantitative mass-loading curve of the WGM biosensor (Figure 3a) showed a large sensor response to the binding reaction, with saturated mass-loading ~4400 pg∙mm^−2^ after 1400 s. After the cDNA binding has come to completion and no further significant WGM sensor shift signals are observed, fresh pH 8.0 Tris-EDTA-MgCl_2_ buffer was placed in the O-ring droplet cell. Next, we added 2 µL of 100 µM Hg^2+^ aptamer stock solution, upon which the WGM sensor signal sharply increased a second time and equilibrated at ~2000 pg∙mm^−2^ additional mass-loading, as the DNA hybridization reaction reaches equilibrium in about 150 s (Figure 3b). This result indicates that ~45.5% of the cDNA immobilized on the microsphere sensor is accessible for hybridization.

### 4.2. Sensitivity for Hg^2+^ Detection

The microsphere functionalized for the DNA strand displacement reaction was then exposed to various concentrations of Hg^2+^ ions, ranging from 50 nM to 1 µM. For this step, pH 8.0 Tris-MgCl_2_ buffer was utilized in order to prevent the possible interferences from the metal chelator EDTA. In response to the Hg^2+^ ions, the 3′-terminal fragment of the aptamer displaced the cDNA in binding to 5′-terminal bases of the Hg^2+^ aptamer by the formation of the intra-strand T-Hg-T complex at the sites of T-T mismatches [33] (Figure 2). As a result, the aptamer strand was then released from the microsphere in hairpin conformation. 

Figure 4a shows the saturated mass-unloading changes found upon the onset of the DNA strand displacement reaction by different concentrations of Hg^2+^ ions. In the presence of 1 µM Hg^2+^ ions, the system demonstrated a quick response, reaching saturated equilibrium, −846.7 ± 11.5 pg∙mm^−2^ (mean ± standard deviation), in about 10 min. As the absolute total mass-loading was observed to be smaller than the one recorded for the binding of the aptamer, this indicated that the aptamer was not completely released from the surface, perhaps due to unspecific interactions that occurred at the surface or due to the unsuccessful release of the aptamer by the strand displacement reaction. As we decreased the Hg^2+^ concentration, the system response speed became slower because fewer aptamers were displaced in a certain time window. We found a linear relationship between the total mass-unloading and Hg^2+^ concentration as shown in Figure 4b, which allowed for a simple quantification of our sensor response. The detection capability of our sensing system was demonstrated at ~50 nM Hg^2+^, with a final mass-unloading of −74.4 ± 10.3 pg∙mm^−2^. Note that each aptamer has seven Hg^2+^ binding sites, with a maximum of three binding sites directly participating in the DNA strand displacement reaction. The closed hairpin hybridizes and displaces the 5′-ACAA-3′ region of the 10 mer cDNA fragment (5′-biotin-ACAAACATGA-3′). The other four binding sites participate in the formation of the hairpin structure by self-hybridization of the aptamer strand [1]. We have also investigated the sensor response to lower Hg^2+^ concentrations such as 10 nM and 5 nM. In these experiments, however, weak shift signals were observed (data not shown), suggesting a ~5 nM detection limit in our proof-of-principle experiments that will need further optimization for trace metal ion analysis. 

Next, we have investigated the relationship between the initial rate of the DNA strand displacement reaction and the Hg^2+^ concentration (Figure 4c). The initial absolute mass-unloading rate of each reaction in Figure 4a was determined by a linear fit to data points within the initial two-minute time interval. We have observed an increase of the absolute aptamer mass-unloading rate with an increase in the Hg^2+^ concentration. This result indicated that the kinetics of a DNA strand displacement reaction that utilizes a DNA hairpin can be controlled by varying the concentration of the ionic species that bind to an aptamer sequence in the hairpin.

### 4.3. Selectivity of Mercury Detection

To evaluate the selectivity of our system, eight common metal ions (Cu^2+^, K^+^, Mg^2+^, Na^+^, Ni^2+^, Pb^2+^, Zn^2+^, and Ca^2+^) were tested under the same assay conditions as Hg^2+^. All experiments were conducted three times, with reproducible results as shown in Figure 5. The final absolute mass-loading response of 1 µM Hg^2+^ was 846.7 ± 11.5 pg∙mm^−2^. For the other metal ions, the final absolute mass-loadings were recorded at much smaller values. The largest absolute mass-unloading signal was recorded for calcium ions at ~47.0 ± 13.1 pg∙mm^−2^, only about one-eighteenth of the signal that we recorded for Hg^2+^ under these conditions. These results confirmed a high specificity for the detection of Hg^2+^ using strand displacement on a WGM sensor system.

## 5. Conclusions

We have demonstrated in proof-of-principle experiments a label-free method for determining the concentration of Hg^2+^ ions using a DNA strand displacement reaction integrated on a WGM sensor. We showed a reproducible detection capability of ~50 nM Hg^2+^ ions, and a linear sensor response in a concentration range from 50 nM to 1 μM Hg^2+^ ions. Our system exhibited more than 18-fold greater selectivity for Hg^2+^ over other common metal ions such as Ni^2+^, Pb^2+^, and Zn^2+^. In comparison to established label-free techniques for sensitive Hg^2+^ detection [34,35,36], the WGM detection scheme represented a novel approach for detecting low molecular weight ionic species on a WGM sensor using DNA nanotechnology. With further improvements of the WGM technique itself and refinements of DNA strand displacement reactions in particular, our proof-of-principle study may aid in the future development of highly sensitive and specific Hg^2+^ detection schemes that operate in complex media and on chip-scale devices and require only minimal sample volumes. Additionally, our results also indicated that the kinetics of a DNA strand displacement reaction based on hairpin conformation can be controlled by varying the concentration of an ionic species that binds to an aptamer, offering yet another strategy to control this kind of reaction. In summary, in this work we developed a novel label-free platform for specific Hg^2+^ detection. Similar detection strategies based upon strand displacement reactions and plasmon-enhanced WGM sensors that operate at the single-molecule level may show promise for a variety of detection tasks.
